# A log-Weibull spatial scan statistic for time to event data

**DOI:** 10.1186/s12942-018-0137-9

**Published:** 2018-06-13

**Authors:** Iram Usman, Rhonda J. Rosychuk

**Affiliations:** 1 Department of Pediatrics, 3-077, Edmonton Clinic Health Academy, University of Alberta, 11405 87 Avenue NW, Edmonton, AB T6G 1C9 Canada; 2 Department of Pediatrics, 3-524, Edmonton Clinic Health Academy, University of Alberta, 11405 87 Avenue NW, Edmonton, AB T6G 1C9 Canada

**Keywords:** Spatial scan statistic, Log-Weibull distribution, Time to event, Atrial fibrillation and flutter, Emergency department

## Abstract

**Background:**

Spatial scan statistics have been used for the identification of geographic clusters of elevated numbers of cases of a condition such as disease outbreaks. These statistics accompanied by the appropriate distribution can also identify geographic areas with either longer or shorter time to events. Other authors have proposed the spatial scan statistics based on the exponential and Weibull distributions.

**Results:**

We propose the log-Weibull as an alternative distribution for the spatial scan statistic for time to events data and compare and contrast the log-Weibull and Weibull distributions through simulation studies. The effect of type I differential censoring and power have been investigated through simulated data. Methods are also illustrated on time to specialist visit data for discharged patients presenting to emergency departments for atrial fibrillation and flutter in Alberta during 2010–2011. We found northern regions of Alberta had longer times to specialist visit than other areas.

**Conclusions:**

We proposed the spatial scan statistic for the log-Weibull distribution as a new approach for detecting spatial clusters for time to event data. The simulation studies suggest that the test performs well for log-Weibull data.

## Background

The existence of more than presumed numbers of cases of a disease condition in a geographic region is referred to as a spatial disease cluster. Timely detection of spatial disease clusters enables health authorities to better understand the distribution of disease and if possible, control disease. A large number of methods have been proposed and applied by authors for the identification and evaluation of geographical disease clusters and disease surveillance, and the spatial scan statistics (SSS) is one of them.

The SSS, with its possible extensions has been widely used as a standardized approach for the last two decades, not only in the disease clustering but also in various other fields of study like natural disasters [1], forestry [2], astronomical data [3], history [4], and psychology [5]. It was first proposed by Kulldorff and Nagarwalla and has the capability of identifying spatial clusters of variable sizes and locations [6]. The key reasons for the popularity of this method include that it identifies the cluster location and tests the tendency to cluster [7]. According to Costa and Assunção, the latter advantage is considered to be more important in terms of health related interventions than global clustering results [7]. The SSS’s based on the Bernoulli and Poisson models are frequently used for count data for cluster identification and geographical disease surveillance [8, 9]. These scan statistics have been further extended to other kinds of data such as ordinal [11], multinomial [12], continuous [13], and correlated count data [14].

Time to event data along with the censoring component (e.g., survival data) is one of the important health outcomes for which the SSS is of interest [9]. The SSS for time to event data is used to determine if there are geographical clusters with either longer than expected and/or shorter than expected time to event. The exponential [9] and Weibull [10] SSS’s (adjusted for censoring) have already been developed for time to event data. We propose the log-Weibull as an alternative distribution for the SSS for cluster detection of time to event data. The log-Weibull distribution has wide applications in extreme value theory. Our focus is to establish a new SSS for the detection of rare and extreme events.

In the Methods section, we describe the existing Weibull SSS and the newly developed SSS based on the log-Weibull distribution. The Application section contains the results from the identification of clusters of longer times to specialist follow-up after an emergency department presentation for atrial fibrillation and flutter in Alberta, Canada. Simulation studies are performed to investigate power, the effect of right (type I) differential censoring, and ability to identify the true cluster by the log-Weibull and Weibull spatial scan statistics.

## Methods

The SSS identifies the geographic zones from a study region that have the strongest indication of representing a spatial cluster. It uses data such as administrative health data collected for geographical sub-regions, each characterized by a centroid (population or geographic based). The SSS imposes a circular searching window of radius *r* on each centroid with its center at the coordinate of a centroid [6]. A zone (Z) defined by this circular window is comprised of all the individuals in the sub-regions whose centroids lie inside the circle [6]. For the purpose of the analysis, an upper bound r*  is chosen for the radius of the circular window [10]. For each region’s centroid, its nearest neighbours covering altogether *r* * percent of the total population are calculated. For any given position of the centroid, the radius of the window is expanded continuously to take any value between 0 and r*  [10]. During the expansion, every time a new zone is created with an inclusion of a new neighbouring centroid in the circular window [14]. Zones defined in this way have irregular geographical boundaries depending on the size and shape of those sub-regions, whose centroids lie inside the spatial scan window [14].

The methodology of the SSS is based on calculating the maximum log likelihood ratio (LLR). The SSS partitions the geographical area into zones (i.e., areas of potential cluster versus the rest of the study region) and the LLR is calculated every time when a new zone is created for each centroid [8, 10]. The zone maximizing the LLR is called the primary (most likely) cluster. Let the primary cluster be the zone $\hat{Z}$ that maximizes the LLR. The hypothesis under consideration is:

H_0_: The disease risk is constant over $\hat{Z} \cup \hat{Z}^{c}$ vs. H_1_: There is an elevated risk in $\hat{Z}$.

Let $G$ be the whole study region which can be partitioned into $Z$ and $Z^{c}$ mutually exclusive sub-regions, where Z indicates a zone designated to be a potential cluster and $Z^{c}$ is the rest of the study region. Let $N = n_{in} + n_{out}$ be the total number of individuals in $G$, where $n_{in}$ and $n_{out}$ are the total individuals inside and outside the zone, respectively. The subscripts “*in*” and “*out*” indicate that the objects are calculated from the individuals inside and outside the zone, respectively.

Let the *i*th individual have a time to event $T_{i} ,\,(i = 1,\ldots,N)$ or a fixed right censoring time $L_{i}$. The event time $T_{i}$ is observed if $T_{i} \le L_{i} (\delta_{i} = 1)$, and $L_{i}$ is observed if $T_{i} > L_{i} (\delta_{i} = 0)$, where $\delta_{i}$ is the indicator to represent if time is censored or not [9]. The observed time is defined as $t_{i} = \hbox{min} (T_{i} ,L_{i} )$. Let $R = r_{in} + r_{out}$ be the total number of uncensored observations, where $r_{in}$ and $r_{out}$ are the total number of uncensored observations inside and outside the zones, respectively. These are defined as $r_{in} = \sum\nolimits_{i \in Z} {\delta_{i} }$ and $r_{out} = \sum\nolimits_{{i \in Z^{c} }} {\delta_{i} }$.

### Weibull distribution

Bhatt and Tiwari established the SSS based on the Weibull distribution. The Weibull model is a nice generalization of the exponential model that includes a shape parameter with the existing scale parameter [10]. The additional parameter provides the opportunity to the Weibull hazard function to take different shapes rather than to be a constant. We provide a brief summary of the methodology, complete details can be found in the paper presented by Bhatt and Tiwari [10]. Let the times to event $T_{i} 's,\,(i = 1,\ldots,N)$ be i.i.d. with the Weibull probability density function (PDF) $f\left( {T_{i} } \right) = \frac{1}{\theta }pT_{i}^{{\left( {p - 1} \right)}} e^{{\left( {{{ - T_{i}^{p} } \mathord{\left/ {\vphantom {{ - T_{i}^{p} } \theta }} \right. \kern-0pt} \theta }} \right)}}$, where $\theta$ and $p$ are the scale and shape parameters, respectively. Let the time to event for each individual inside the zone be distributed as the Weibull distribution with $\theta_{in}$ and $p_{in}$ as the scale and shape parameters, respectively. Similarly, assume that the times to event for individuals outside the zone are Weibull distributed with $\theta_{out}$ and $p_{out}$ as the scale and shape parameters, respectively. The null hypothesis under consideration is $H_{0} :\theta_{in} = \theta_{out}$ versus the alternative hypotheses $H_{1} :\theta_{in} < \theta_{out}$, $H_{1} :\theta_{in} > \theta_{out}$, or $H_{1} :\theta_{in} \ne \theta_{out}$. The alternative hypotheses show that at least one zone is detected with either shorter than expected, longer than expected, or simultaneously both longer and shorter than expected times to events. The likelihood ratio test statistic for the Weibull SSS for $H_{1} :\theta_{in} \ne \theta_{out}$ is

$$\lambda = \mathop {\hbox{max} }\limits_{Z} \frac{{\left( {\frac{R}{{\sum\nolimits_{i \in G} {t_{i}^{p} } }}} \right)^{R} }}{{\left( {\frac{{r_{in} }}{{\sum\nolimits_{i \in Z} {t_{i}^{{p_{in} }} } }}} \right)^{{r_{in} }} \left( {\frac{{r_{out} }}{{\sum\nolimits_{{i \in Z^{c} }} {t_{i}^{{p_{out} }} } }}} \right)^{{r_{out} }} }}.$$

For $H_{1} :\theta_{in} < \theta_{out}$, $\lambda$ is multiplied by $I\left( {\frac{{r_{in} }}{{\sum\nolimits_{i \in Z} {t_{i}^{p} } }} < \frac{{r_{out} }}{{\sum\nolimits_{{i \in Z^{c} }} {t_{i}^{p} } }}} \right)$ , and similarly for $H_{1} :\theta_{in} > \theta_{out}$, it is multiplied by $I\left( {\frac{{r_{in} }}{{\sum\nolimits_{i \in Z} {t_{i}^{p} } }} > \frac{{r_{out} }}{{\sum\nolimits_{{i \in Z^{c} }} {t_{i}^{p} } }}} \right)$.

### Log-Weibull distribution

The log-Weibull distribution is a specialized case of the generalized extreme value distribution. It is often used to model the distribution of extreme values, strength, event history data such as quick wear-out after reaching a certain age, and logarithms of times [17]. We assume that times to event $T_{i} 's,\,(i = 1,\ldots,N)$ are independently and identically distributed (i.i.d.) with the log-Weibull PDF $f\left( {T_{i} } \right) = \frac{1}{b}\exp \left( {\frac{{T_{i} - a}}{b}} \right)\exp \left\{ { - \exp \left( {\frac{{T_{i} - a}}{b}} \right)} \right\}$ , where $a$ and $b$ are the location and scale parameters, respectively. The survival function for the log-Weibull distribution is $S\left( {T_{i} } \right) = \exp \left\{ { - \exp \left( {\frac{{T_{i} - a}}{b}} \right)} \right\}.$

Let the time to event for each individual inside zone *Z* be log-Weibull distributed with $a_{in}$ and $b_{in}$ as the location and scale parameters, respectively. Similarly, the time to event for each individual outside zone $Z$(i.e., inside $Z^{c}$) follows the log-Weibull distribution with $a_{out}$ and $b_{out}$ as the location and scale parameters, respectively. The null hypothesis $H_{0} :b_{in} = b_{out}$ for any $Z$ is contrasted with one of three alternative hypotheses: $H_{1} :b_{in} < b_{out}$, $H_{1} :b_{in} > b_{out}$, or $H_{1} :b_{in} \ne b_{out}$. The likelihood function $L\left( Z \right) = L\left( {Z,b_{in} ,b_{out} } \right)$ for the log-Weibull SSS can be written as:

$$\begin{aligned} L\left( Z \right) & = \prod\limits_{i \in Z} {\left[ {\left( {f\left( {T_{i} } \right)} \right)^{{\delta_{i} }} \left( {S\left( {L_{i} } \right)} \right)^{{1 - \delta_{i} }} } \right]} \prod\limits_{{i \in Z^{c} }} {\left[ {\left( {f\left( {T_{i} } \right)} \right)^{{\delta_{i} }} \left( {S\left( {L_{i} } \right)} \right)^{{1 - \delta_{i} }} } \right]} \\ & = \prod\limits_{i \in Z} {\left[ {\left( {\frac{1}{{b_{in} }}e^{{\left( {\frac{{T_{i} - a_{in} }}{{b_{in} }}} \right) - e^{{\left( {\frac{{T_{i} - a_{in} }}{{b_{in} }}} \right)}} }} } \right)^{{\delta_{i} }} \left( {e^{{ - e^{{\left( {\frac{{L_{i} - a_{in} }}{{b_{in} }}} \right)}} }} } \right)^{{1 - \delta_{i} }} } \right]} \, \times \prod\limits_{{i \in Z^{c} }} {\left[ {\left( {\frac{1}{{b_{out} }}e^{{\left( {\frac{{T_{i} - a_{out} }}{{b_{out} }}} \right) - e^{{\left( {\frac{{T_{i} - a_{out} }}{{b_{out} }}} \right)}} }} } \right)^{{\delta_{i} }} \left( {e^{{ - e^{{\left( {\frac{{L_{i} - a_{out} }}{{b_{out} }}} \right)}} }} } \right)^{{1 - \delta_{i} }} } \right]} \\ & = \left( {b_{in} } \right)^{{ - r_{in} }} \left( {b_{out} } \right)^{{ - r_{out} }} e^{{\left( {\sum\limits_{i \in Z} {\delta_{i} \left( {\frac{{t_{i} - a_{in} }}{{b_{in} }}} \right) - \sum\limits_{i \in Z} {e^{{\left( {\frac{{t_{i} - a_{in} }}{{b_{in} }}} \right)}} } } } \right)}} e^{{\left( {\sum\limits_{{i \in Z^{c} }} {\delta_{i} \left( {\frac{{t_{i} - a_{out} }}{{b_{out} }}} \right) - \sum\limits_{{i \in Z^{c} }} {e^{{\left( {\frac{{t_{i} - a_{out} }}{{b_{out} }}} \right)}} } } } \right)}} \\ \end{aligned}$$

Taking the natural log on both sides, we have

$$\ln L\left( Z \right) = - r_{in} \ln b_{in} - r_{out} \ln b_{out} + \sum\limits_{i \in Z} {\delta_{i} \left( {\frac{{t_{i} - a_{in} }}{{b_{in} }}} \right) - } \sum\limits_{i \in Z} {e^{{\left( {\frac{{t_{i} - a_{in} }}{{b_{in} }}} \right)}} } + \sum\limits_{{i \in Z^{c} }} {\delta_{i} \left( {\frac{{t_{i} - a_{out} }}{{b_{out} }}} \right) - } \sum\limits_{{i \in Z^{c} }} {e^{{\left( {\frac{{t_{i} - a_{out} }}{{b_{out} }}} \right)}} }$$

For $H_{1} :b_{in} \ne b_{out}$, for at least one zone $Z$, the corresponding likelihood ratio statistic is

$$\lambda = \frac{{\max_{{Z,b_{{in \ne b_{out} }} }} L\left( {Z,b_{in} ,b_{out} } \right)}}{{\max_{{Z,b_{{in = b_{out} }} }} L\left( {Z,b_{in} ,b_{out} } \right)}} = \frac{{L\left( {\hat{Z}} \right)}}{{\hat{L}}}$$

where $\hat{Z}$ is the zone maximizing $L\left( {Z,b_{in} ,b_{out} } \right)$ under $H_{1}$, and $\hat{L}$ is the maximum of $L\left( {Z,b_{in} ,b_{out} } \right)$ under $H_{0}$. The maximum likelihood estimators (MLE’s) of the parameters $b_{in} ,\,b_{out} ,\,a_{in} ,$ and $a_{out}$ for any arbitrary zone $Z$ can be obtained by the following equations,

$$\begin{aligned} \frac{\partial \ln L\left( Z \right)}{{\partial b_{in} }} & = - \frac{{r_{in} }}{{b_{in} }} - \frac{1}{{b_{in}^{2} }}\sum\limits_{i \in Z} {\delta_{i} \left( {t_{i} - a_{in} } \right) - \sum\limits_{i \in Z} {e^{{\left( {\frac{{t_{i} - a_{in} }}{{b_{in} }}} \right)}} } \left( {\frac{ - 1}{{b_{in}^{2} }}\left( {t_{i} - a_{in} } \right)} \right)} = 0 \\ \frac{\partial \ln L\left( Z \right)}{{\partial b_{out} }} & = - \frac{{r_{out} }}{{b_{out} }} - \frac{1}{{b_{out}^{2} }}\sum\limits_{{i \in Z^{c} }} {\delta_{i} \left( {t_{i} - a_{out} } \right) - \sum\limits_{{i \in Z^{c} }} {e^{{\left( {\frac{{t_{i} - a_{out} }}{{b_{out} }}} \right)}} } \left( {\frac{ - 1}{{b_{out}^{2} }}\left( {t_{i} - a_{out} } \right)} \right)} = 0 \\ \end{aligned}$$

$$\begin{aligned} \frac{\partial \ln L\left( Z \right)}{{\partial a_{in} }} & = \frac{1}{{b_{in} }}\sum\limits_{i \in Z} {\left( { - \delta_{i} } \right) - \sum\limits_{i \in Z} {e^{{\left( {\frac{{t_{i} - a_{in} }}{{b_{in} }}} \right)}} } \left( {\frac{ - 1}{{b_{in} }}} \right)} = 0 \\ \frac{\partial \ln L\left( Z \right)}{{\partial a_{out} }} & = \frac{1}{{b_{out} }}\sum\limits_{{i \in Z^{c} }} {\left( { - \delta_{i} } \right) - \sum\limits_{{i \in Z^{c} }} {e^{{\left( {\frac{{t_{i} - a_{out} }}{{b_{out} }}} \right)}} } \left( {\frac{ - 1}{{b_{out} }}} \right)} = 0 \\ \end{aligned}$$

Thus the MLE’s of the scale parameters $b_{in}$ and $b_{out}$ are

$$\hat{b}_{in} = \frac{1}{{r_{in} }}\sum\limits_{i \in Z} {\left( {t_{i} - \hat{a}_{in} } \right)} \left[ {e^{{\left( {\frac{{t_{i} - \hat{a}_{in} }}{{\hat{b}_{in} }}} \right)}} - \delta_{i} } \right]$$ and $$\hat{b}_{out} = \frac{1}{{r_{out} }}\sum\limits_{{i \in Z^{c} }} {\left( {t_{i} - \hat{a}_{out} } \right)} \left[ {e^{{\left( {\frac{{t_{i} - \hat{a}_{out} }}{{\hat{b}_{out} }}} \right)}} - \delta_{i} } \right]$$, respectively.

Similarly, the MLE’s of the location parameters $a_{in}$ and $a_{out}$ are obtained by the equations $r_{in} = \sum\limits_{i \in Z} {e^{{\left( {\frac{{t_{i} - \hat{a}_{in} }}{{\hat{b}_{in} }}} \right)}} }$ and $r_{out} = \sum\limits_{{i \in Z^{c} }} {e^{{\left( {\frac{{t_{i} - \hat{a}_{out} }}{{\hat{b}_{out} }}} \right)}} }$, respectively.

Under $H_{1} :b_{in} \ne b_{out}$, the obtained MLE’s provide

$$L\left( {\hat{Z}} \right) = \left( {\hat{b}_{in} } \right)^{{ - r_{in} }} \left( {\hat{b}_{out} } \right)^{{ - r_{out} }} e^{{\left( {\sum\limits_{i \in Z} {\delta_{i} \left( {\frac{{t_{i} - \hat{a}_{in} }}{{\hat{b}_{in} }}} \right) + \sum\limits_{{i \in Z^{c} }} {\delta_{i} \left( {\frac{{t_{i} - \hat{a}_{out} }}{{\hat{b}_{out} }}} \right)} } } \right)}} e^{ - R} .$$

Similarly, under $$H_{0} :b_{in} = b_{out}$$,

$$\hat{L} = \left( {\hat{b}_{G} } \right)^{ - R} e^{{\left( {\sum\limits_{i \in G} {\delta_{i} \left( {\frac{{t_{i} - \hat{a}_{G} }}{{\hat{b}_{G} }}} \right)} } \right)}} e^{ - R} .$$

So, the likelihood ratio statistic for $H_{1} :b_{in} \ne b_{out}$ is

$$\lambda = \frac{{\max_{Z} \left( {\hat{b}_{in} } \right)^{{ - r_{in} }} \left( {\hat{b}_{out} } \right)^{{ - r_{out} }} e^{{\left( {\sum\limits_{i \in Z} {\delta_{i} \left( {\frac{{t_{i} - \hat{a}_{in} }}{{\hat{b}_{in} }}} \right) + \sum\limits_{{i \in Z^{c} }} {\delta_{i} \left( {\frac{{t_{i} - \hat{a}_{out} }}{{\hat{b}_{out} }}} \right)} } } \right)}} }}{{\left( {\hat{b}_{G} } \right)^{ - R} e^{{\left( {\sum\limits_{i \in G} {\delta_{i} \left( {\frac{{t_{i} - \hat{a}_{G} }}{{\hat{b}_{G} }}} \right)} } \right)}} }}.$$

In order to address the alternative hypotheses $b_{in} < b_{out}$ and $b_{in} > b_{out}$, the function $\lambda$ is multiplied by $I\left( {\hat{b}_{in} < \hat{b}_{out} } \right)$ and $I\left( {\hat{b}_{in} > \hat{b}_{out} } \right)$, respectively.

### Permutation test procedure

Since there is no closed analytical form of the distribution of the test statistic $\lambda$, a permutation test procedure is used to test the statistical inference of the selected clusters. The exact distribution of the time to events is unknown and it is not possible to generate the simulated data under the null hypothesis. To overcome this situation, the observed pairs $\left\{ {\left( {t_{i} ,\delta_{i} } \right),i = 1,2, \ldots ,N} \right\}$ are permuted 999 times among the individual geographical coordinates of the original study region [9]. For each permuted dataset, the log-likelihood is calculated for each zone and the most likely cluster preserving the maximum log-likelihood in the dataset is saved. A *p* value is calculated as the fraction of permutations that are at least as extreme as the test statistic from the observed time to event data [18]. This permutation step ensures that no matter how the observed time to event data are distributed, this distribution is preserved for each permuted dataset. This factor provides valid statistical inference since all the permuted datasets are equally distributed [9]. Secondary clusters are the significant spatial clusters that do not overlap with the primary cluster [9]. These clusters are ranked with their corresponding LLR values and the associated *p* values are calculated by comparing the *k*th (say) highest likelihood in the real dataset with the maximum likelihood in the randomly permuted datasets [9]. Note that the use of a permutation test procedure means that there will be variation in the exact *p* values for successive analyses of the same datasets.

## Results

### Emergency data application

**Fig. 1:**
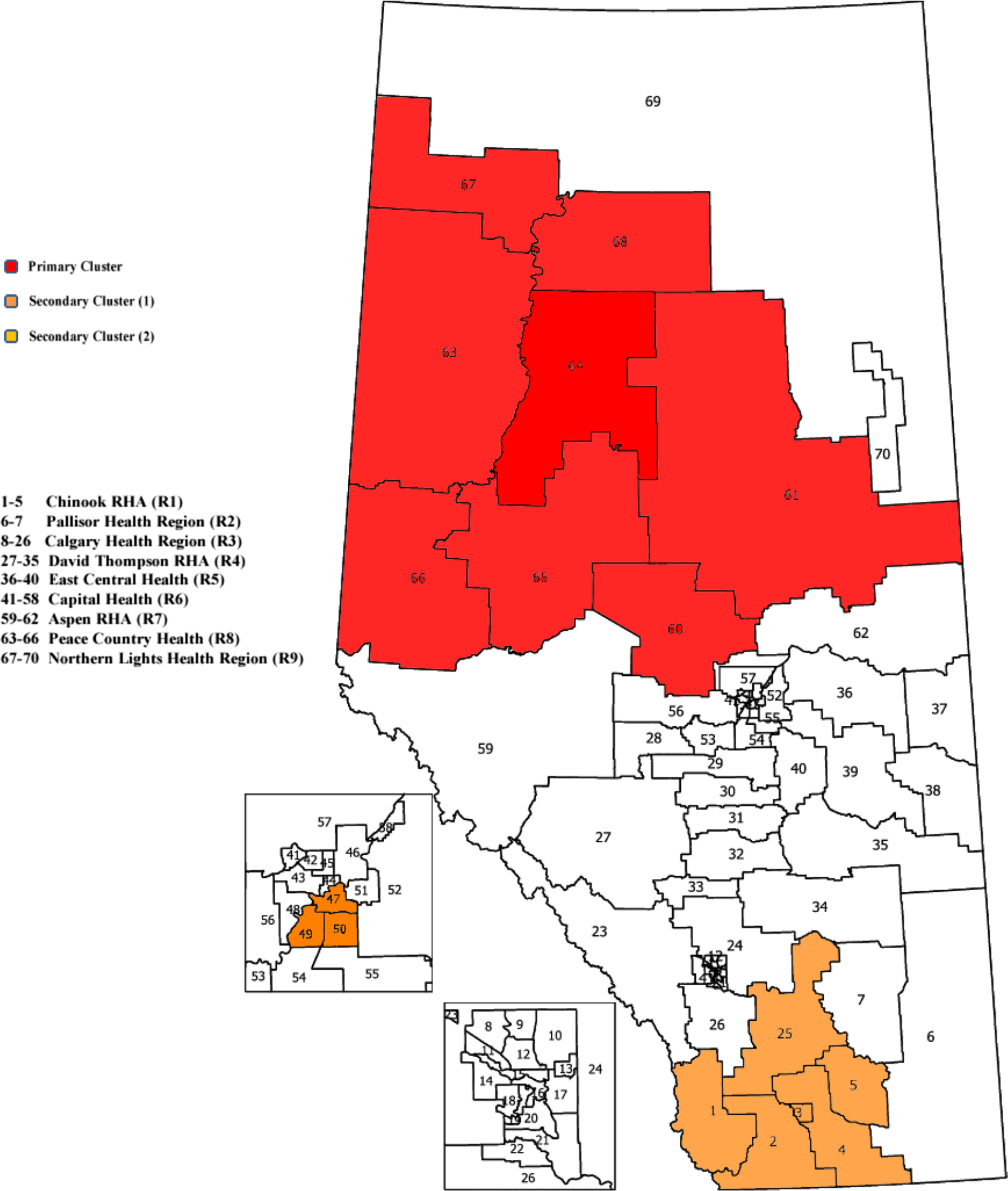
Alberta map highlighting the primary and secondary clusters for the log-Weibull spatial scan statistic

We illustrate the log-Weibull SSS on population based administrative data (age ≥ 35) for patients discharged from the emergency department (ED) who presented with atrial fibrillation and flutter (AFF) in the province of Alberta during April 1, 2010, to March 31, 2011. In 2003, the province of Alberta was divided into nine administrative health areas also called Regional Health Authorities (RHAs) [19]. These RHA’s were further partitioned into 70 sub-Regional Health Authorities (sRHAs) (Fig. 1, numbered 1–70). The sRHAs have diverse population sizes ranging from 550 to 140,211 with a median population size of 46,075 in 2011 and are the smallest geographical units available for analysis. For each sRHA’s centroid based on population, the latitude and longitude of the centroids are provided by Alberta Health [19]. Distances between the pairs of sRHA population-based centroids are ordered and used to create the nearest neighbours.

The key outcome of interest is the time from ED discharge for AFF to the 1st specialist visit during 365 days of the study period. The specialist in this study is considered as a cardiology (CARD) or internal medicine (INMD). A specialist follow-up visit can occur between ED end time, to the end of the study. Each discharged ED presentation during April 1, 2010, to March 31, 2011, with a follow-up visit to the specialist during its ED end time, to March 31, 2011 is considered a complete time to event outcome. If the patient did not have specialist visit by the end of the study (March 31, 2011), the outcome is referred to as right (type-I) censored. Each Alberta resident making at least one discharged ED presentation for AFF during the fiscal year is referred to as a case (patient).

The methodology used in this study does not adjust for repeated ED presentations of cases. Hence, independent patient data is considered by taking only the last ED visit out of the multiple visits. The calculations are performed using the R and S-Plus [20, 21]. Each cluster can contain only a maximum of $r* = 10\%$ of the study population. The variable scanning windows are created for each sRHA to absorb neighbours up to 10% of the total population. This upper bound is chosen based on the feasibility of analysis and time restrictions. There are about 1.95 M adults in the study population, among them the discharged subset is comprised of 3039 cases (30% censored, 54% male) with an average age of 68.04 years. The median time to event for the whole dataset is 81 days and the corresponding 95% confidence interval (CI) is 76–86 days.

**Fig. 2:**
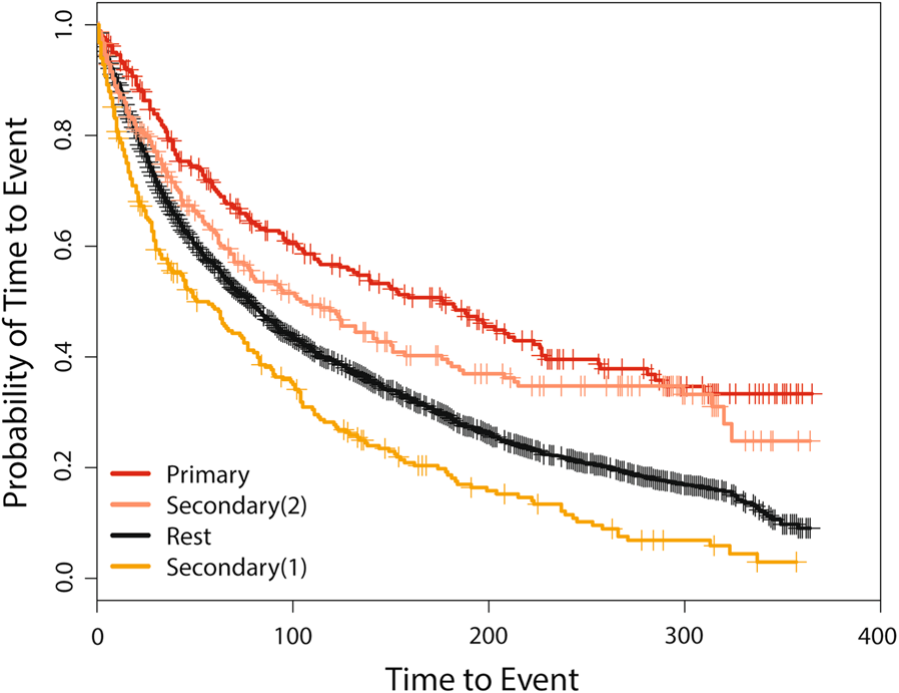
Kaplan Meier curves for the detected primary and secondary clusters and rest of the province for time to first specialist visit for the log-Weibull spatial scan statistic

The identified primary and secondary clusters are shown in Table 1 and Fig. 1. The most likely cluster with significantly longer times to events is mainly from R7-R9 RHAs. This cluster is identified with 260 observed number of cases. The LLR is 710.75 with the associated *p* value (*P*) of 0.001. This SSS provides two different statistically significant secondary clusters. The first one is a part of R6 and the second cluster is a combination of sRHAs from R1 and R3. Median times to event are 177, 51, and 104 days for inside the primary, secondary (1), and secondary (2) detected clusters, respectively. The corresponding 95% CI’s are 128–223, 38–75, and 77–150 days. For the entire province, collectively excluding the primary and both secondary clusters, the median event time is 78 days and the 95% CI is (71, 84) days. Figure 2 shows the Kaplan–Meier curves for the detected primary and secondary clusters and the rest of the province. The SSS based on the Weibull distribution has also been applied to the same Alberta Health data, and is capable of detecting the same primary cluster as of the log-Weibull distribution i.e., from R7-R9 RHA’s, with no significant secondary cluster.

**Table 1:** Spatial scan results for the log-Weibull distribution

Cluster	sRHA	Population	Cases	LLR	P
Primary	64 65 68 63 60 67 66 61	124,094	260	710.75	0.001
Secondary (1)	50 47 49	175,893	249	423.27	0.001
Secondary (2)	2 3 4 1 5 25	99,425	239	394.08	0.001

### Simulation studies

Simulation studies are conducted to investigate the power of detecting a potential cluster and the effect of right differential censoring on cluster detection. All of the datasets are analyzed with the log-Weibull and Weibull SSS’s. Time to event data are randomly generated for 500 individuals with five different probability models: the exponential, Weibull, log-Normal, gamma, and log-Weibull. The Alberta geography is used as the geography for analysis and the Alberta population is used to create the zones for the simulation studies. Like the spatial scan analysis of the real administrative data, an upper bound of 10% is imposed on the population size.

For all simulated datasets, a true cluster of 25 individuals is created at a subregion of R201 sRHA, to have longer time to events than the rest of the province. This subregion was chosen because it was rural and away from the detected rural cluster in the real Alberta ED data. R201 was assigned the same percentage of individuals as of the real dataset (i.e., approximately 5% cases in each simulated data). This choice was feasible for simulation studies to run in a reasonable amount of time. Right differential censoring is added with the ratios of 20%:20%, 20%:40%, and 40%:20% for inside:outside the true cluster. For example, 20%:40% means that 20% censoring is used within the true cluster and 40% outside the true cluster.

One thousand simulated datasets are generated from the probability models defined above using the differential censoring settings under the alternative hypotheses of the existence of longer than expected time to event clusters. The choice of 1000 simulations is the same as what was chosen for the development of the Weibull SSS [10] and was computationally timely. For symmetry, parameters for each probability model are chosen in such a way that they provide a constant mean of 2 outside the true cluster and means of 10, 15, and 20 inside the true cluster for each censoring ratio. These values were chosen to be similar to the inside:outside times to event means ratio from real data used in the application.

For each simulated dataset, 999 random permutations are performed to get the *p* values from the permutation testing procedure. Let, $Z^*,Z^{(m)} ,$ and $M$ represent the true cluster, the cluster identified in the *m*th simulations, and total number of simulations, respectively. Power is calculated as the proportion of datasets out of 1000 having *p* values < 0.05 [9, 10], not necessarily detecting the true cluster i.e.,

$${\text{Power}} = \frac{1}{M}\sum\limits_{m = 1}^{M} {I_{{\left[ {Z^{\left( m \right)} ;P\left( {Z^{\left( m \right)} } \right) < 0.05} \right]}} } .$$

In order to observe the strength of identification of the true cluster by each SSS, three different proportions are calculated for mutually exclusive situations from 1000 randomly generated datasets under each probability model for all censoring situations. These indicators are essentially the same as those reported for the exponential and Weibull based SSS’s [9, 10], and we have adapted slightly to reflect the aggregate nature of the data.

These are the proportion of datasets:

1. Perfectly identifying the true cluster $\left( {{\text{PI}} = \frac{1}{M}\sum\limits_{m = 1}^{M} {I_{{\left[ {Z^* = Z^{\left( m \right)} } \right]}} } } \right)$;
2. Identifying a large cluster including the true cluster $\left( {{\text{LC}} = \frac{1}{M}\sum\limits_{m = 1}^{M} {I_{{\left[ {Z^* \subset Z^{\left( m \right)} } \right]}} } } \right)$; and,
3. Not identifying the true cluster $\left( {{\text{NI}} = \frac{1}{M}\sum\limits_{m = 1}^{M} {I_{{\left[ {Z^*{ \varsubsetneq }Z^{\left( m \right)} } \right]}} } } \right)$.

In addition to the three cluster performance measures listed above, a global indicator for performance assessment has been used [22] based on the coefficient developed by Tanimoto [23, 24]. The Tanimoto coefficient (TC) is computed for each simulated data set and measures the similarity between a simulated and detected cluster by using the ratio of the intersecting cluster cohort to the union cluster cohort. In order to calculate TC, four types of spatial units (SUs) are calculated and defined as:

- True Positive (TP) = SUs both within $Z^{*}$ and $Z^{(m)}$;
- False Positive (FP) = SUs only within $Z^{(m)}$;
- False Negative (FN) = SUs only within $Z^{*}$; and,
- True Negative (TN) = SUs not within either cluster.

The TC computed for each simulated data set is $TC = \frac{TP}{TP + FP + FN}$. The geographical region used in this simulation study is divided into 70 SUs. When no significant cluster is detected i.e., *p* value is higher than 0.05, we get TP = 0, FP = 0, TN = 69, and FN = 1.

The average Tanimoto coefficient (TC_a_) and the cumulated Tanimoto coefficient (TC_c_) were used as the statistics of TC. These are defined as $TC_{a} = \frac{1}{M}\sum\limits_{m = 1}^{M} {\frac{{TP_{m} }}{{\left( {TP_{m} + FP_{m} + FN_{m} } \right)}}}$ and $TC_{c} = \frac{{\sum\limits_{m = 1}^{M} {TP_{m} } }}{{\sum\limits_{m = 1}^{M} {\left( {TP_{m} + FP_{m} + FN_{m} } \right)} }}$. Global performance is assessed using TC_a_ and TC_c_ by taking both location accuracy and power into account at the same time. Guttmann et al. have assessed the superiority of TC_c_ over TC_a_ based on their functional properties and variability, and observed that TC_c_ has more power of capturing low accuracy in cluster location [22].

**Fig. 3:**
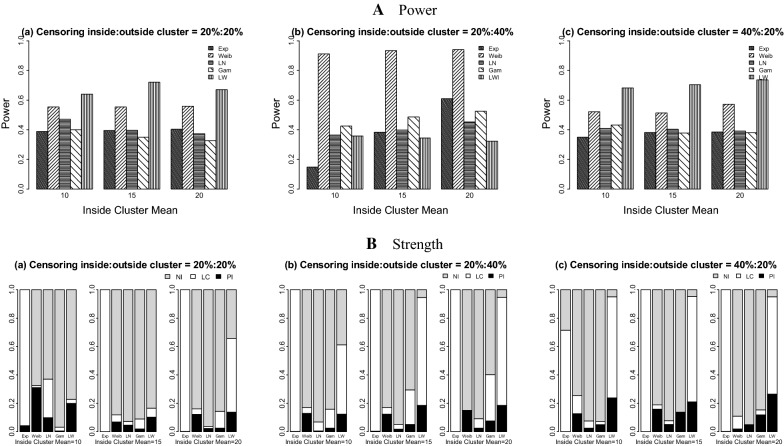
Power and strength of the log-Weibull spatial scan statistic for cluster detection under right differential censoring. Datasets are generated using five probability models with outside cluster mean = 2. *PI* perfect identification, *LC* large cluster (including true cluster), *NI* no identification, *Exp* exponential, *Weib* Weibull, *LN* log-normal, *Gam* Gamma, *LW* log-Weibull

**Fig. 4:**
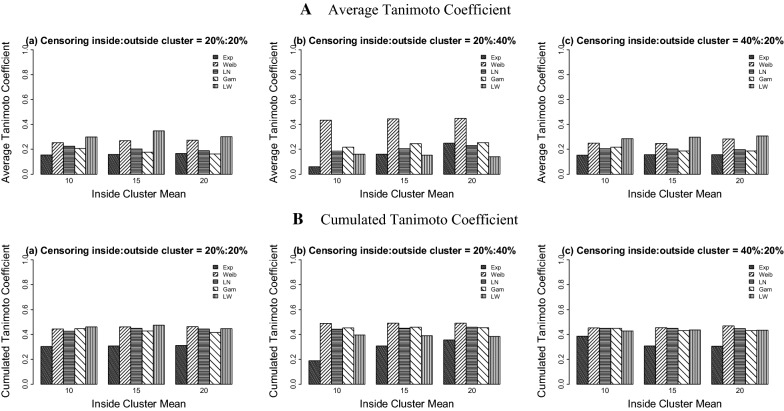
Average and cumulated Tanimoto coefficients of the log-Weibull spatial scan statistic for cluster detection under right differential censoring. Datasets are generated using five probability models with outside cluster mean = 2

Using the log-Weibull SSS (Table 2, Figs. 3 and 4), the results show that the values of power vary from 0.326 to 0.721 for the 20%:20% censoring, from 0.148 to 0.941 for the 20%:40% censoring situation, and range from 0.350 to 0.737 for the 40%:20% censoring case. Overall, the maximum power is seen when the data are generated under the Weibull distribution and the minimum power is observed for the datasets distributed with the gamma and exponential probability models.

**Table 2:** Simulation study results for the log-Weibull spatial scan statistic

Data distribution	IC		Power			PI			LC			TC_a_			TC_c_		
	M	V	a	b	c	a	b	c	a	b	c	a	b	c	a	b	c
Exponential	10	100.0	0.388	0.148	0.350	0.042	0.001	0.000	0.958	0.999	0.714	0.155	0.060	0.153	0.304	0.189	0.386
	15	225.0	0.395	0.383	0.381	0.000	0.003	0.000	1.000	0.997	1.000	0.158	0.160	0.156	0.307	0.308	0.308
	20	400.0	0.403	0.609	0.385	0.002	0.000	0.002	0.998	1.000	0.998	0.166	0.248	0.157	0.312	0.356	0.306
Weibull	10	4.0	0.554	0.913	0.522	0.310	0.128	0.127	0.014	0.041	0.127	0.252	0.435	0.248	0.444	0.489	0.452
	15	10.0	0.554	0.934	0.513	0.069	0.124	0.158	0.049	0.045	0.030	0.270	0.445	0.247	0.461	0.490	0.455
	20	7.0	0.559	0.941	0.573	0.122	0.148	0.020	0.039	0.001	0.089	0.274	0.448	0.283	0.462	0.491	0.468
Log-Normal	10	4.0	0.471	0.364	0.408	0.099	0.005	0.024	0.272	0.064	0.052	0.225	0.185	0.204	0.426	0.442	0.449
	15	10.0	0.397	0.398	0.404	0.046	0.017	0.051	0.026	0.034	0.026	0.203	0.205	0.202	0.449	0.451	0.448
	20	17.0	0.373	0.452	0.391	0.022	0.025	0.049	0.015	0.066	0.000	0.189	0.231	0.196	0.445	0.458	0.447
Gamma	10	5.0	0.400	0.425	0.432	0.005	0.025	0.050	0.027	0.134	0.021	0.207	0.216	0.217	0.446	0.453	0.448
	15	7.5	0.349	0.486	0.378	0.019	0.051	0.138	0.071	0.243	0.000	0.176	0.244	0.186	0.429	0.459	0.431
	20	10.0	0.326	0.525	0.380	0.025	0.076	0.118	0.118	0.326	0.035	0.163	0.253	0.186	0.416	0.454	0.430
Log-Weibull	10	5.5	0.641	0.357	0.682	0.199	0.123	0.238	0.029	0.490	0.714	0.299	0.160	0.286	0.460	0.397	0.429
	15	6.0	0.721	0.344	0.705	0.103	0.186	0.209	0.062	0.760	0.744	0.347	0.152	0.298	0.474	0.389	0.436
	20	6.5	0.670	0.323	0.737	0.138	0.186	0.264	0.518	0.762	0.688	0.302	0.141	0.307	0.446	0.384	0.434

Five probability models each with three different means inside true cluster are used under three right censoring cases: *a* = 20%:20%, *b* = 20%:40%, *c* = 40%:20% outside cluster: mean = 2; variance = 4(Exponential), 0.188(Weibull), 2(log-Normal), 1(Gamma), and 5(log-Weibull). *IC* inside cluster, *M* mean, *V* variance, *PI* perfect Identification, *LC* large cluster identification, *TC*_*a*_ average Tanimoto coefficient, *TC*_*c*_ cumulated Tanimoto coefficient

The proportions of datasets perfectly identifying the true cluster fluctuate for the log-Weibull SSS. They are between 0.000 and 0.310 for the 20%:20% case, range from 0.000 to 0.186 for the 20%:40% censoring ratio, and are between 0.000 and 0.264 for the 40%:20% censoring setting, respectively. Under the large cluster identification cohort for the log-Weibull distribution, there are high proportions of the true cluster detected. These proportions range from 0.000 to 1.000 for all three differential censoring situations. Overall, the maximum proportion of perfect identification is achieved for the datasets generated from the log-Weibull distribution. The datasets from the exponential distribution have the highest proportions of large cluster identification including the true cluster among all five probability models. A few decreases are found in the power and the strength of identification of the true cluster for each model, when comparing the 20%:20% to the 20%:40% and 40%:20% censoring cases.

For the log-Weibull SSS, the values of TC_a_ range from 0.060 to 0.448 for all three censoring situations. The TC_c_ values lie between 0.189 and 0.491 with very less variability among the five probability models used to generate the data.

**Fig. 5:**
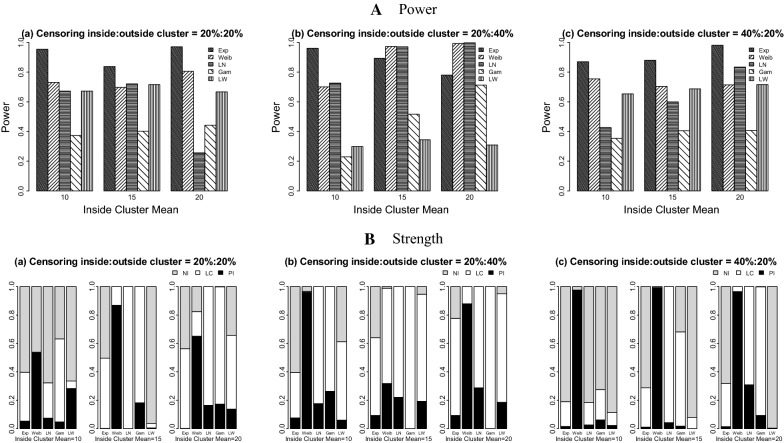
Power and strength of the Weibull spatial scan statistic for cluster detection under right differential censoring. Datasets are generated using five probability models with outside cluster mean = 2. *PI* perfect identification, *LC* large cluster (including true cluster), *NI* no identification, *Exp* exponential, *Weib* Weibull, *LN* log-normal, *Gam* Gamma, *LW* log-Weibull

**Fig. 6:**
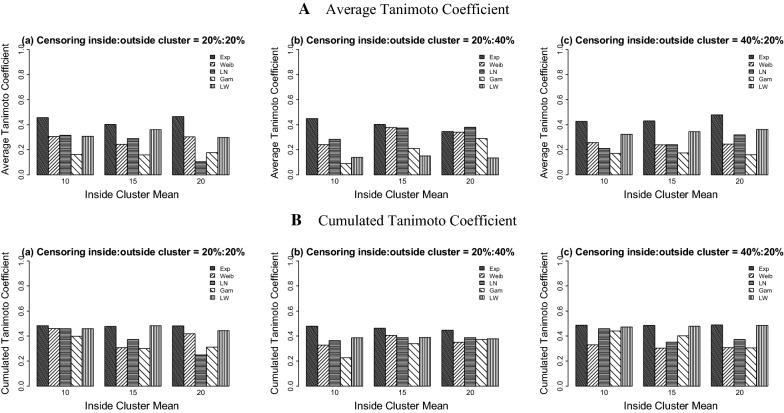
Average and cumulated Tanimoto coefficients of the Weibull spatial scan statistic for cluster detection under right differential censoring. Datasets are generated using five probability models with outside cluster mean = 2

For the Weibull SSS (Table 3, Figs. 5 and 6), the overall results for the power and all the proportions’ performances of the datasets are less variable than the results of the log-Weibull SSS. The power values of detecting a potential cluster are between 0.256 and 0.971 for the 20%:20% censoring setting, range from 0.230 to 0.999 for the 20%:40% censoring ratio, and are between 0.355 and 0.981 for the 40%:20% case. The proportions of perfectly detecting a true cluster are high for all three censoring situations across all of the datasets as compared to the log-Weibull distribution, being least for the exponential model. The non-zero proportions of datasets generated under five probability distributions who do not identify the true cluster are between 0.000 and 0.997. The power values increase as the difference between the means of inside and outside the cluster increase and similar effects are seen for the strength of detection of the true cluster.

**Table 3:** Simulation study results for the Weibull spatial scan statistic

Data distribution	IC		Power			PI			LC			TC_a_			TC_c_		
	M	V	a	b	c	a	b	c	a	b	c	a	b	c	a	b	c
Exponential	10	100.0	0.954	0.962	0.871	0.052	0.076	0.015	0.345	0.320	0.175	0.456	0.449	0.427	0.483	0.479	0.487
	15	225.0	0.838	0.894	0.881	0.001	0.094	0.011	0.494	0.547	0.276	0.403	0.403	0.431	0.476	0.462	0.485
	20	400.0	0.971	0.781	0.981	0.001	0.094	0.014	0.562	0.682	0.306	0.465	0.346	0.478	0.481	0.446	0.489
Weibull	10	4.0	0.732	0.701	0.755	0.538	0.961	0.976	0.000	0.006	0.000	0.306	0.240	0.258	0.461	0.327	0.329
	15	10.0	0.697	0.973	0.704	0.869	0.317	0.993	0.131	0.672	0.000	0.243	0.378	0.238	0.308	0.407	0.303
	20	7.0	0.806	0.993	0.715	0.652	0.879	0.966	0.172	0.121	0.034	0.304	0.340	0.245	0.418	0.349	0.307
Log-Normal	10	4.0	0.672	0.726	0.427	0.074	0.176	0.027	0.248	0.824	0.156	0.315	0.283	0.211	0.458	0.363	0.459
	15	10.0	0.721	0.971	0.599	0.000	0.221	0.043	1.000	0.779	0.957	0.290	0.374	0.240	0.372	0.388	0.352
	20	17.0	0.256	0.999	0.835	0.164	0.287	0.309	0.836	0.713	0.691	0.105	0.380	0.320	0.248	0.387	0.371
Gamma	10	5.0	0.373	0.230	0.355	0.048	0.263	0.062	0.584	0.737	0.214	0.163	0.090	0.171	0.398	0.226	0.440
	15	7.5	0.401	0.517	0.405	0.181	0.000	0.018	0.819	1.000	0.664	0.158	0.210	0.175	0.300	0.339	0.403
	20	10.0	0.443	0.713	0.406	0.173	0.000	0.093	0.826	1.000	0.906	0.176	0.289	0.161	0.312	0.371	0.306
Log-Weibull	10	5.5	0.672	0.298	0.654	0.282	0.059	0.022	0.054	0.553	0.091	0.308	0.138	0.323	0.458	0.385	0.472
	15	6.0	0.717	0.344	0.688	0.005	0.192	0.000	0.031	0.754	0.080	0.360	0.150	0.343	0.482	0.387	0.478
	20	6.5	0.668	0.309	0.716	0.138	0.185	0.001	0.518	0.764	0.002	0.297	0.135	0.362	0.443	0.377	0.484

Five probability models each with three different means inside true cluster are used under three right censoring cases: *a* = 20%:20%, *b* = 20%:40%, *c* = 40%:20% outside cluster: mean = 2; variance = 4(Exponential), 0.188(Weibull), 2(log-Normal), 1(Gamma), and 5(log-Weibull). *IC* inside cluster, *M* mean, *V* variance, *PI* perfect identification, *LC* large cluster, identification *TC*_*a*_ average Tanimoto coefficient, *TC*_*c*_ cumulated Tanimoto coefficient

For the Weibull SSS, the values of TC_a_ and TC_c_ range from 0.090 to 0.478 and 0.226 to 0.489, respectively. This study shows that the Weibull SSS has more similar results for the spatial cluster detection based on power, proportions of cluster detection and global detection test regardless of the probability model used for the data generation, whereas the performance of the log-Weibull SSS is best when the datasets are generated from the log-Weibull distribution.

## Discussion

The spatial scan statistic (SSS) is a widely used statistical technique for the identification of the spatial clusters of different data types by using various probability distributions. In the context of time to event data, the SSS has the ability to detect geographical clusters of cases with either longer and/or shorter than expected event times. These clusters can be adjusted for censoring, if the appropriate probability model is used.

We have proposed the SSS for the log-Weibull distribution as a new approach for detecting spatial clusters for time to event data. The log-Weibull distribution has wide applications in extreme value theory for modeling extreme and rare events. The new log-Weibull method and the Weibull SSS are applied to administrative data from Alberta Health consisting of time from ED discharge for an AFF presentation to 1st specialist visit within 365 days in Alberta during 2010–2011. Results from the SSS show that the primary cluster is detected at the Peace Country, Northern Lights, and Aspen regional Health Authorities. The most likely cluster is comprised of rural areas in northern Alberta which have sparse or low population and have further distances to major metropolitan centres. The results suggest that people living in these northern rural areas may not have regular or quick access to the follow-up care to a specialist after an ED presentation. Our results are in agreement with the recognized issue of health care access for rural residents and strategies such as mobile services, telehealth, and rotating specialists have been suggested and/or implemented [25]. While we recognize that the censoring might be quite early for the patients with an ED visit in late 2011 and the methods may be effected by short follow-up, the effects would be across all areas of the province and we feel that the results are likely linked to real clustering and are plausible given the recognized issue of health care access.

The simulation studies indicate that the power of detecting the potential cluster is higher for the 20%:20% censoring ratio as compared to the 20%:40% and 40%:20% settings. This comparison is also true in the context of identification of a true cluster. When either the Weibull or log-Weibull distributions is used for the SSS, the effect of the right differential censoring on power and detection of the true cluster is similar. For both of the probability models used under the SSS’s, as the difference between means of time to event data increase inside and outside the true cluster, the power and proportion of detection of the true cluster also increase. It can be observed from the overall results of both SSS’s that the Weibull SSS has good power for detecting a potential cluster for the datasets distributed with any of the five probability models used in this study. However, overall the log-Weibull SSS’s performance is satisfactory for the data distributed as the log-Weibull. For the identification of the true cluster, the Weibull SSS shows less variability on the simulated datasets than the log-Weibull SSS. The log-Weibull SSS shows the most power to detect a true cluster for the datasets generated from the log-Weibull distribution. When various differential censoring situations are considered, the global performance indicators for the log-Weibull SSS do not vary widely. Conversely, when there was less censoring inside the cluster than outside the cluster, the log-Weibull SSS had highly variable performance that depended on the underlying data distribution.

The results based on the global indicator for performance assessment also support the above conclusions, identifying that the Weibull SSS detects the true cluster with more power and location accuracy both at the same time, whereas the log-Weibull SSS shows high significant cluster detection accuracy for the datasets generated from log-Weibull probability distribution. It is also observed that the log-Weibull distribution has a good ability to detect a broader cluster including the true cluster instead of identifying exact true cluster. It is suggested that the log-Weibull SSS can be used to detect a spatial cluster for the time to event data distributed as log-Weibull. Based on the simulation study results for both SSSs, the log-Weibull SSS proved to be less effective than the Weibull SSS when the dataset is generated from the exponential distribution. When the underlying data distribution is not exponential, the log-Weibull SSS has slightly reduced performance than the Weibull SSS; however, the log-Weibull SSS had similar performance across different underlying data distributions, especially when the censoring ratio is higher inside the true cluster than outside the true cluster.

There are many opportunities for future work. For example, the proposed methodology based on the SSS for the log-Weibull distribution does not adjust for important factors such as age and gender. In future, such covariates can be adjusted in the analysis of the identification of potential clusters for time to event data. Furthermore, the new developed method can only be performed on a purely spatial setting. The space–time scan statistic has been developed by other authors in both retrospective [15] and prospective [16] ways. In the future, the SSS based on the log-Weibull distribution can be extended to the space–time setting, and similar simulation studies can be performed to investigate power of detection of space–time clusters.

## Conclusions

We have proposed a new SSS using the log-Weibull distribution. The new method has been applied to specialist follow-up data in Alberta, and the SSS’s have been compared and contrasted for time to event data generated from simulations. The simulation studies suggest that the SSS based on the log-Weibull distribution performs well for log-Weibull data. The log-Weibull distribution, being a specialized case of the generalized extreme value distribution, has a wide application in extreme value theory for modeling extreme and rare events.

## Abbreviations

AFF: atrial fibrillation and flutter
CARD: cardiology
CI: confidence interval
ED: emergency department
INMD: internal medicine
LLR: log likelihood ratio
MLE’s: maximum likelihood estimators
P: *p* value
PDF: probability density function
RHAs: Regional Health Authorities
SSS: spatial scan statistics
sRHAs: sub-Regional Health Authorities
PI: perfect identification
LC: large cluster identification
NI: no identification
TP: true positive
FP: false positive
FN: false negative
TN: true negative
TC_a_: average Tanimoto coefficient
TC_c_: cumulated Tanimoto coefficient
